# Terminological Confusion About Sedation in Palliative Care: Results of an International Online Vignette Survey

**DOI:** 10.1089/jpm.2023.0159

**Published:** 2024-04-02

**Authors:** Alexander Kremling, Claudia Bausewein, Carsten Klein, Stephan Nadolny, Christoph Ostgathe, Eva Schildmann, Kerstin Ziegler, Jan Schildmann

**Affiliations:** ^1^ Interdisciplinary Center for Health Sciences, Institute for History and Ethics of Medicine, Martin Luther University Halle-Wittenberg, Halle, Germany.; ^2^ Department of Palliative Medicine, LMU University Hospital, Ludwig Maximilian University München, Comprehensive Cancer Centre Munich (CCCM), Germany.; ^3^ Department of Palliative Medicine, Friedrich-Alexander-University Erlangen-Nürnberg (FAU), Comprehensive Cancer Center Erlangen-EMN (CCCER-EMN), Universitätsklinikum Erlangen, Erlangen, Germany; ^4^ Institute for Educational and Health-Care Research in the Health Sector, Hochschule Bielefeld—University of Applied Sciences and Arts, Germany.; ^5^ Palliative Medicine, Faculty of Medicine, University of Augsburg, Germany.; ^6^ Department of Criminal Law, Criminal Procedural Law, Commercial Criminal Law and Medical Criminal Law, Friedrich-Alexander-University Erlangen-Nürnberg (FAU), Erlangen, Germany.

**Keywords:** cross-sectional survey, deep sedation, palliative care, palliative sedation, terminology, vignette study

## Abstract

**Background:**

Terminological problems concerning sedation in palliative care and consequences for research and clinical decision making have been reported frequently.

**Objectives:**

To gather data on the application of definitions of sedation practices in palliative care to clinical cases and to analyze implications for high-quality definitions.

**Design:**

We conducted an online survey with a convenience sample of international experts involved in the development of guidelines on sedation in palliative care and members of the European Association for Palliative Care (EAPC). Participants were asked to apply four published definitions to four case vignettes. Data were analyzed using descriptive statistics.

**Results:**

A total of 32 experts and 271 EAPC members completed the survey. The definitions were applied correctly in *n* = 2200/4848 cases (45.4%). The mean number of correct applications of the definitions (4 points max.) was 2.2 ± 1.14 for the definition of the SedPall study group, 1.8 ± 1.03 for the EAPC definition, 1.7 ± 0.98 for the definition of the Norwegian Medical Association, and 1.6 ± 1.01 for the definition of the Japanese Society of Palliative Medicine. The rate of correct applications for the 16 vignette-definition pairs varied between 70/303 (23.1%) and 227/303 (74.9%). The content of definitions and vignettes together with free-text comments explains participants' decisions and misunderstandings.

**Conclusions:**

Definitions of sedation in palliative care are frequently incorrectly applied to clinical case scenarios under simplified conditions. This suggests that clinical communication and research might be negatively influenced by misunderstandings and inconsistent labeling or reporting of data.

Clinical Trial Registration Number: DRKS00015047.

## Background

Terminological problems concerning sedation in palliative care have been frequently reported: authors have discussed terminological confusion as a plausible cause of strikingly variable frequencies of the practice, which have been reported in empirical studies,^1–3^ called for more uniform definitions,^4–7^ and debated the neutrality of terms.^8–10^ We identified 29 definitions differing in the term defined as well as the content and structure of the definition in a recent systematic literature review of definitions in guidelines on sedation in palliative care.^11^

Studies with case vignettes in Germany demonstrated considerable differences in the intuitive attribution of the most commonly used term “palliative sedation.”^12^ A retrospective chart review revealed “no consistent pattern regarding labelling.”^13^ A qualitative study indicated that the term “palliative sedation” is used inconsistently and sometimes misinterpreted as deep continuous sedation.^14^ This might impede decision making and transparent communication with patients and within care teams.

What counts as a, for example, “palliative sedation” depends on the logical inclusion and exclusion criteria in its definition. What counts as a “good palliative sedation,” that is, one that fulfils standards of good palliative care, depends on practical requirements care that can be stated separately, for example, in guidelines. Sometimes, practical requirements such as refractoriness or intolerability of suffering are part of the definition. The content of the definitions varies in this respect.^11^ We wanted to know how well professionals can identify these differences and how this affects the way they apply them to cases.

Problems in applying specific definitions have not been studied so far, despite plausible hypotheses, and terminological decisions or misunderstandings have not been attributed to the content of the definitions on an empirical basis. There are no data on the false/correct application of definitions and it is not clear which parts of which definitions are problematic. The evidence on problems of applying definitions to clinical cases is necessary to develop better concepts and conceptually sound study designs. Good evidence could help to identify better definitions, highlight specific problems of definitions, and indicate measures to improve conceptual understanding for practice and research.

Therefore, the aim of this study was to determine how well experts involved in the development of guidelines on sedation in palliative care and members of the European Association for Palliative Care (EAPC) can apply definitions of sedation practices in palliative care to concrete clinical case vignettes and to compare different definitions and cases.

## Methods

### Study design

We conducted a vignette-based, cross-sectional online survey among experts on sedation in palliative care and EAPC members from July to August 2021.

### Questionnaire

#### Definitions

We selected four definitions to cover definitional options and keep the length of the survey manageable (Table 1): the definition published by the Norwegian Medical Association represents reduced and simple definitions. It includes only the sedative effect, an intention (to alleviate suffering) and one “good practice” restriction (refractory nature of the suffering).^15^ Second, we chose the definition provided by the EAPC, since it is the most prominent and part of the EAPC's framework for the development of guidelines on sedation.^16^ Third, we chose the Japanese definition reported by Morita et al. because it was published recently and explicitly formulated to resolve confusion about intentions.^17^ Fourth, we developed a definitional paragraph according to a terminology table consented in the SedPall project (“From anxiolysis to deep continuous sedation—the development of a recommendation for sedation in specialized palliative care”). This terminology was constructed explicitly to reduce previously identified misunderstandings by a step-by-step descriptive definition of key terms.^18^

**Table 1.** Definitions

Source	Definition
Norway^15^	By palliative sedation is meant pharmacological depression of the level of consciousness to alleviate suffering that cannot be relieved in any other way.
European Association for Palliative Care^16^	Therapeutic (or palliative) sedation in the context of palliative medicine is the monitored use of medications intended to induce a state of decreased or absent awareness (unconsciousness) to relieve the burden of otherwise intractable suffering in a manner that is ethically acceptable to the patient, family, and health care providers.
Japan^17^	Palliative sedation is defined as “administration of sedatives for the purpose of alleviating refractory suffering.” It does not depend on whether the physician intends to reduce the patient's consciousness.
SedPall study group (Germany)^18^	Intentional sedation to alleviate suffering: sedation is the process or result of inducing a state of consciousness scoring <0 ( = below normal alertness) on the Richmond Agitation-Sedation Scale modified for palliative care inpatients (RASS-PAL) scale. It is intentional if it is used as a means to achieve a previously defined treatment goal. It is performed to alleviate suffering when the treatment goal is to alleviate a patient's suffering.

#### Vignettes

We followed published guidance for vignette construction^19,20^ and used four published case vignettes as starting points.^12,21,22^ An expert panel of four members of the SedPall research group with expertise in oncology, palliative medicine, medical ethics, and philosophy adapted the vignettes concerning clinical plausibility and the possibility of an unambiguous answer regarding the (non-)presence of treatment as defined in the four definitions. We adapted the vignettes until consensus was achieved regarding the correct answers (Table 4) according to the wording of the definition, that is, independent of recommendations for good practice (Table 2).

**Table 2.** Vignettes and Rationale for Selection

	Vignette
1	A patient's unmanageable nausea is treated with 2.5 mg lorazepam every eight hours. The patient is sleepy due to this medication. The patient wakes up when being addressed during visits from her family. The team attending does not set up any further monitoring. The life expectancy of the patient is estimated to be hours to a few days. The patient states that she is still feeling nauseous during two attempts to reduce the dose. In response, the dose is increased again to the initial 2.5 mg after consultation with the patient, so that she would not feel the nausea because of a lower state of vigilance. The patient dies a few hours after the medication has been adjusted. *Rationale: In vignette 1, a low dose of lorazepam which, nevertheless, has a sedative effect is finally administered with the intention of reducing consciousness of the patient to treat unmanageable nausea but without any monitoring measures installed. We wanted to know whether the small dose would influence the decisions.*
2	A 60-year-old patient had been suffering from cerebral corticobasal degeneration for 1 year. He was bedridden and incontinent at admission to the palliative care unit. Furthermore, he had dysarthria with restricted ability to communicate and visual impairment. He complained of pain and suffered from his immobility and loss of autonomy, resulting in a state of depressed mood. An adjustment of relaxant physiotherapy and pain medication showed good effects, however, treatment with antidepressants did not. The patient became calmer and opened up after several conversations. He, nevertheless, described his suffering as being unbearable. A physician applied midazolam (5 mg/h) without administering artificial hydration or nutrition, intending to help by making the patient less aware of his situation. The patient slept deeply with this medication, and his condition was monitored regularly. The treatment with midazolam was discontinued at the insistence of the surprised family. The patient awoke and expressed his displeasure that nobody had consulted him about this kind of procedure and that he, at least, would have wanted to say goodbye to his family. *Rationale: In vignette 2, we presented a case of clinical misconduct. The sedative effect is induced intentionally in a situation of refractory and severe suffering, but without the patient's consent. The sedation is stopped after intervention of the family. We wanted to know how the misconduct influences the decisions, especially because the EAPC definition formulates ethical acceptability as a definitional element, whereas the other definitions are neutral in this respect.*
3	A palliative care team was called in by the treating hemato-oncological team to care for a 58-year-old man with relapsed acute myeloid leukemia after his second bone marrow transplant. He developed a graft versus host disease and a hepatic fungal infection. His course was further complicated by chronic intestinal bleeding. The patient suffered from severe pain despite treatment with morphine. He acknowledged his poor prognosis and asked what could be done to ease his suffering. As a result of a joint team decision and consultation with the relatives, no further attempts with a promising alternative pain medication were carried out. Instead, midazolam was applied at a dose of up to 4 mg/h. The patient was sleeping quietly under this medication and was monitored regularly. *Rationale: In vignette 3, we presented a case where a viable treatment option is omitted but the patient was intentionally sedated anyway. Since refractoriness is part of most definitions and used in three of our chosen definitions, we wanted to know whether participants would decide accordingly.*
4	A 75-year-old female patient with an ulcerating carcinoma at the base of her mouth and mild dementia with signs of delirium was admitted to the palliative care unit because of “exacerbation of pain and unrest.” She claimed to be in great pain, felt nauseous, and showed signs of unrest. Specific attempts targeted at her respective symptoms were carried out separately at the beginning of her treatment. Neuroleptic medication did not have the desired effect. After receiving her and her relatives' agreement, it was attempted to treat her unrest with midazolam (1 mg/h) without negatively influencing her ability to communicate. The dosage was increased to 2 mg/h. Paradoxically, her unrest increased to a point of agitation. The treatment with midazolam was stopped immediately and alternative treatment options were discussed. *Rationale: In vignette 4, we presented a case of paradox agitation following administration of midazolam. We wanted to know whether participants noticed that there is no sedative effect at all and especially whether they noticed that the Japanese definition only requires the “administration of sedatives” without mentioning a sedative effect of the medication.*

EAPC, European Association of Palliative Care.

**Table 4.** Correct Answers per Definition (4 Point Max)

Groups	Norwegian definition		EAPC definition		Japanese definition		SedPall definition	
	Mean ± SD	Median (min–max)	Mean ± SD	Median (min–max)	Mean ± SD	Median (min–max)	Mean ± SD	Median (min–max)
Expert group (*n* = 32)	2.1 ± 0.98	2 (0–4)	2.0 ± 1.11	2 (0–4)	1.8 ± 0.81	2 (0–3)	2.2 ± 1.11	2 (0–4)
EAPC member group (*n* = 271)	1.7 ± 0.98	2 (0–4)	1.8 ± 1.02	2 (0–4)	1.5 ± 1.03	1 (0–4)	2.2 ± 1.15	2 (0–4)
Total (*n* = 303)	1.7 ± 0.98	2 (0–4)	1.8 ± 1.03	2 (0–4)	1.6 ± 1.01	1 (0–4)	2.2 ± 1.14	2 (0–4)

Min–Max, minimum to maximum value; SD, standard deviation.

#### Questions

The survey consisted of 24 nonrandomized, nonadaptive questions on 17 pages (8 on one page to describe the sample plus one page for each terminological question, see the Supplementary Appendix). Eight sociodemographic questions were asked concerning age, sex, country, profession, professional experience in years, setting of work, and experience in (1) research in palliative care and (2) research on sedation in palliative care. Participants were asked to apply each of the four definitions to each of the four vignettes and decide whether the vignette represented a case of the sedation term defined, resulting in 16 questions, which we announced at the beginning of the survey (Supplementary Appendix). We did not provide information about the source of the definitions or connected recommendations for practice, so decisions had to be made based on the wording of the definition only.

Answers were rated on a nominal level:

- Yes, this is a case of palliative sedation according to the definition above.
- No, this is not a case of palliative sedation according to the definition above.
- I'm not sure whether this is a case of palliative sedation according to the definition above.

A mail address was provided to contact AK for questions or remarks.

Participants could provide a free-text comment on each decision and change previous answers via a back button until submission. A completeness check was not possible. A cookie was placed to prevent double participation after submission—no IP check or further log file analysis was undertaken. The survey was created with LimeSurvey, running on servers of the Martin Luther University. The questionnaire was pretested internally by the SedPall study group and members of the associated departments for usability, technical functionality, and clarity of vignettes, questions and answers provided. No adjustments were necessary. Only replies with at least one answer were stored. Calculation of the recruitment rate was, thus, not possible.

### Sampling and administration

We used different contact modes for experts involved in guideline development on sedation in palliative care and members of the EAPC via convenience sampling in both groups.

#### Expert sample

The sampling frame was the set of authors identified in a systematic review of definitions in guidelines on the topic.^11^ Every author or (if unknown) associated institutions were contacted and invited to participate. We asked for contact information about coauthors and ongoing guideline or revision projects. Experts were asked not to participate in the EAPC survey if they were also members of the EAPC to avoid double participation. Finally, we sent out 169 individual invitations with anonymized single access tokens (closed survey). After completing the survey, an error message was displayed in case of a renewed access.

#### EAPC members' sample

Participants consisted of individual members of the national associations listed on the EAPC website (individual members of each EAPC membership association are associated members of the EAPC).^23^ Due to our research focus, we excluded pediatric associations. We also excluded German and Austrian associations, because we conducted a separate survey in German. The chairs or chief executive officers of the associations were contacted and invited to distribute the survey. We asked for a member count. The link was distributed by the societies via mailing lists or in a newsletter (open survey). A reminder was sent to both groups after two and three weeks.

### Data processing and analysis

We did not intend a representative sample and generalizable conclusions. Therefore, we did not conduct an *a priori* power analysis or apply inferential statistical methods. We used descriptive statistics and calculated absolute and relative frequencies instead. We created a dichotomous variable for each vignette-definition pair with the characteristics “correct” and “incorrect.” Questions that were answered with “I am not sure” were assigned to the category “incorrect.”

We calculated mean and standard deviation (SD) for each definition (4 points max), case (4 points max), and overall number of correct answers (four definitions with four questions each: 16 points max) to analyze correct answers. Cross-tabulations were used to examine the results regarding the influence of sociodemographic variables.

We reported the completion rate (Supplementary Appendix) and excluded questionnaires that had missing values in the terminological questions to avoid bias. Consequently, we did not impute missing values or any further statistical correction method. We excluded participants from the open (EAPC) survey who reported that they were not working in palliative care. Analysis was conducted with IBM SPSS Statistics Version 24.

We also analyzed the four vignette-definition pairs with the highest/lowest rate of correct answers qualitatively. One author and one research assistant (AK, KW) paraphrased and grouped comments and analyzed them for possible explanations of the participants' decisions.

### Research ethics

The anonymous, nonmandatory survey was approved by the research ethics committee of the Martin Luther University (no. 2021-019). No IP addresses, cookies, or time stamps were saved. Only one author (AK) had access to the password-protected raw data in LimeSurvey. Study information was provided on the first page of the survey. No incentives were paid.

## Results

A total of 735 participants started the survey. The completion rate was 42.4% (*n* = 312). Consequently, 423 participants did not complete the survey; 166 of these (39.2%) abandoned the survey during the sociodemographic questions; 50/423 (11.8%) participants left the survey after the first vignette-definition pair, and 362/423 left after the first four questions (the first vignette). We did not receive a member count from two societies (Supplementary Appendix).

Of the 312 completed surveys, 303 were included in the analysis. See the Supplementary Appendix for the flow of participants in the expert and EAPC group.

The majority of participants were female, about two-thirds were physicians and the mean age was 51 years. Participants worked predominantly in the hospital and community setting. See Table 3 for detailed sociodemographic data.

**Table 3.** Sociodemographic Characteristics (*n* = 303)

Variable	Values	*n *(%)^a^
Age in years	18–25	1 (0.3)
	26–44	89 (29.4)
	45+	209 (69.0)
	Mean (SD)	50.7 (10.8)
Gender	Female	213 (70.3)
	Male	86 (28.4)
	Diverse	1 (0.3)
Profession	Physician	203 (67.0)
	Nurse	77 (25.4)
	Other	19 (6.3)
	Psychologist	4 (1.3)
Setting	Hospital	161 (53.1)
	Community	122 (40.3)
	Other	16 (5.3)
Professional experience in years	0	4 (1.3)
	1–5	69 (22.8)
	6–10	66 (21.8)
	10+	163 (53.8)
Research experience in palliative care	Yes	172 (56.8)
Research experience in palliative sedation	Yes	73 (24.1)
Expert group	Yes	32 (10.6)

^a^ Due to occasional missing values, 100% may not always be reached.

The overall rate of correct answers was 45.4% (*n* = 2200/4848). The mean number of correct answers per definition (4 points max ± SD) was 1.6 ± 1.01 for Japan, 1.7 ± 0.98 for Norway, 1.8 ± 1.03 for the EAPC, and 2.2 ± 1.14 for SedPall (Table 4). Correct answers for each vignette-definition pair ranged from 84.4% (expert group applying the EAPC definition to vignette 2) to 21.8% (EAPC members applying the EAPC definition to vignette 3). See Table 5 for answers for each vignette-definition pair.

**Table 5.** Answers for Each Vignette-Definition Pair (Correct Answers **Highlighted**)

Vignette	Correct answer (reason for correct negative answer)	Yes (%, experts, EAPC members, total)		Not sure (%)		No (%)	
Norwegian definition							
1	Yes	**50.0**	**46.7**	28.1	18.5	21.9	34.8
		**47.0**		19.5		33.4	
2	Yes	**37.5**	**31.9**	12.5	13.3	50.0	54.8
		**32.5**		13.3		54.3	
3	No (not refractory)	31.3	58.1	12.5	11.9	**56.3**	**30.0**
		55.3		11.9		**32.8**	
4	No (no sedation)	31.3	27.4	6.3	13.3	**62.5**	**59.3**
		27.8		12.6		**59.6**	
EAPC definition							
1	No (no monitoring)	34.4	49.6	31.3	15.2	**34.4**	**35.2**
		48.0		16.9		**35.1**	
2	No (not acceptable)	9.4	17.8	8.0	8.5	**84.4**	**73.7**
		16.9		8.3		**74.8**	
3	No (not refractory)	50.0	70.0	15.6	8.5	**34.4**	**21.5**
		67.9		9.3		**22.8**	
4	No (no intention to sedate)	43.8	40.7	9.4	11.9	**46.9**	**47.4**
		41.1		11.6		**47.4**	
Japanese definition							
1	Yes	**46.9**	**45.2**	28.1	21.9	25.0	33.0
		**45.4**		22.5		32.1	
2	Yes	**34.4**	**31.1**	6.3	20.4	59.4	48.5
		**31.5**		18.9		49.7	
3	No (not refractory)	28.1	46.7	25.0	19.3	**46.9**	**34.1**
		44.7		19.9		**34.4**	
4	Yes	**56.3**	**43.3**	9.4	19.6	34.4	37.0
		**44.7**		18.5		36.8	
SedPall definition							
1	Yes	**53.1**	**54.8**	25.0	20.4	21.9	24.8
		**54.6**		20.9		24.5	
2	Yes	**43.8**	**41.1**	9.4	20.4	46.9	38.5
		**41.4**		19.2		39.4	
3	Yes	**75.0**	**68.9**	12.5	15.2	12.5	15.9
		**69.5**		14.9		15.6	
4	No (no sedative effect)	37.5	31.5	18.8	17.0	**43.8**	**51.5**
		32.1		17.2		**50.7**	

The participants achieved a mean of 7.3 ± 2.64 correct answers across all 16 questions. Mostly minor differences can be seen regarding the sociodemographic data and affiliation to the expert group (Supplementary Appendix). Notable differences exist across all definitions and in the mean number of correct answers between experts and nonexperts (8.1 vs. 7.2), people with no professional experience and more than 10 years of palliative care experience (6.2 vs. 7.3), as well as between nurses and physicians (6.7 vs. 7.6). Members of the expert group who reported that they were not working in palliative care had 5.7 correct answers (compared with 7.5 for those working in an inpatient setting and 7.2 for those working in a home care setting).

### In-depth analysis of high and low performing definition-vignette pairs

We describe the two pairs with the highest rate of correct answers (a, b) and the two pairs with the lowest rate of corrects answers (c, d) in more detail using qualitative data from free text (see the Supplementary Appendix for details): (a) When applying the *EAPC definition to vignette 2*, 74.8% gave the correct answer, which is the highest rate but less than expected given the drastic example of misconduct. Participants identified the violations of good practice in the free-text comments on the “no” and “not sure” decisions as well as the two comments on the “yes” decision.

(b) When applying the SedPall *definition to vignette 4*, 69.5% of participants applied the definition correctly, identifying mostly a “failed attempt” in comments on the (correct) “no” decision as well as the (incorrect) “yes” decision. Participants wondered about the increase of the dosage, the cause of the delirium, and consented treatment goals in the comments on “not sure.” (c) When applying the *EAPC definition to vignette 3*, only 22.8% of the participants (correctly) voted that no “palliative (or therapeutic) sedation” was carried out according to the EAPC, commenting that the symptoms had not been refractory. Participants commented regarding their “not sure” decisions that the symptoms might not be refractory and requested titration. Participants commented on several fulfilled conditions of good practice, such as respect for patient's wishes and monitoring, for the (incorrect) “yes” decision.

(d) When applying the *definition from Norway to vignette 2*, only 32.5% of the participants correctly answered “yes.” Regardless of the correctness of their answer, participants commented on the violations of good practice described, varying between bad communication, nonrefractoriness, and missing consent. When commenting on a (correct) “yes” decision, participants contrasted their comment with the decision, for example, “It meets the definition, but since the patient was not informed, the treatment isn't justified.”

## Discussion

We tested each participant's ability to apply published definitions of guidelines on sedation in palliative care to cases based on the wording of the definition only. The overall rate of correct answers is low and strengthens the concerns raised in the literature about a problematic terminological situation.^12^ The cases were often not labeled correctly on the basis of the wording of a given definition. This is different compared with checking whether a practice satisfies the requirements established in a guideline or personal opinions about good care. Such tasks would have required a different setup of the survey and would probably yield different results.

The range of correct answers for the different pairs demonstrates that overall “performance” of a definition cannot be generalized but depends on the crucial aspects of the vignettes or, in real life, on the aspects of the clinical situation. Some quantitative results for definition-vignette pairs can be explained by the content of the vignette and the definition as well as free-text comments, for example, for the aforementioned pairs (a–d):

**(a)** About three-quarters of the participants gave the correct answer, probably because the EAPC definition includes a condition of ethical acceptability and the vignette describes a violation of patient rights. Nevertheless, even in this case, one out of four participants voted that this is a case of “palliative (or therapeutic) sedation” according to the EAPC or expressed uncertainty. In these two groups, the violation of principles of good care was either not identified or identified but not considered to be relevant to the terminological decision.
**(b)** About 7 out of 10 participants applied the definition correctly, probably because the structure of the definition made clear that the definition requires a sedative effect, whereas in the vignette, paradoxically, agitation is induced.
**(c)** Only every fifth participant voted that no “palliative (or therapeutic) sedation” was carried out according to the EAPC, even though a promising therapeutic option was mentioned, which violates the EAPC definition. The participants seem to have neglected the condition of refractoriness in the definition.
**(d)** Only a third of the participants gave the correct answer, probably because the procedure was assessed as inadequate from an ethical point of view. Participants seem to have implicitly added an ethical condition to the definition, ignoring the fact that the definition is neutral regarding patient consent.

In accordance with our explanation for (b), we think that a plausible explanation for the slightly better results of the SedPall definition generally is that the conditions mentioned in the definition are presented step by step and do not include conditions of good care. This could be one way to realize a descriptive and precise terminology for the spectrum of sedation situations and practices.^6,7^

Narrowing down the best explanations for the participants' answers would require the more detailed testing of case variation (e.g., case 1 with vs. without monitoring). This would most probably result in a lower response and completion rate of the survey. We assume that this would be more possible in an experimental setting with a systematic variation of vignette details.^24^

The survey results strengthen the hypothesis that the terminology concerning sedation in palliative care and particularly its application are problems with a possible negative impact on research and daily practice that needs to be solved.^20^ Terminology should be as simple as possible. One possible strategy is to not include standards of good care into the definition of the treatment but to clearly communicate them as requirements about when to carry out the treatment. This reduces subtleties in the definitions that influence the consistency of their application, as our survey shows. A possible way is a descriptive terminology with a set of precisely defined terms instead of defining a separate treatment entity.^18^ Definitions in new guidelines or future studies should be tested for logical implications, misunderstandings, and consistency of use by the respective target group to reduce negative effects.

Interestingly, 50% of the participants did not complete the survey. This may be explained by the language barrier and the fact that the survey was probably challenging for participants, being more of a test than an attitude poll. Participants who decided to complete the survey were possibly more competent in English language skills and conceptual issues and had a higher motivation to reflect upon the use of terminology than average. This would imply that terminological problems in everyday care could be even greater. At best, the vignettes represent idealized situations with all the relevant information for a correct decision given. In real life, clinical situations are more complex, with possible disagreement about whether symptoms are refractory or unbearable or if changes in the level of consciousness can be attributed to medication.

This makes terminological decisions in everyday practice or retrospective chart reviews even more difficult and increases the risk of varying terminological decisions.

### Limitations

The study population is not representative of all EAPC members or experts. However, quantitative generalizability was not the intention, as we wanted to obtain an initial overview of the topic to see how well participants were performing. We, nevertheless, have a sample, including various countries and different professional groups and levels of experience. As discussed, we cannot rule out a biased sample, but the most probable factors are indicative for even better rates of correct answers in our sample.

We had no possibility to draw a random sample, since we had no access to the EAPC members' sociodemographic or contact details. For this reason, we did not use methods of inferential statistics. In addition, we cannot be certain of the reasons for item- or unit-nonresponse since we do not have any data on this.

Participants might have implicitly added information to the vignettes. We cannot rule out that aspects in the vignettes might still be considered vague, especially regarding refractoriness. We, nevertheless, think that we reduced the possibility of different understandings of the vignettes by our process of vignette construction.

## Conclusions

Terminology should be recognized as a probable source of errors/misunderstandings in decision making, communication, and research. Providing definitions is not sufficient to secure the reliable reporting of data in empirical surveys. New terminology should be accompanied by illustrating material on how to use it. The SedPall definition should be considered a viable terminological alternative.

## Supplementary Material

Supplemental data
