# Supplementary material for: Terminological Confusion About Sedation in Palliative Care: Results of an International Online Vignette Survey
Source: J Palliat Med. 2024 Apr 2;27(4):487–94. doi: 10.1089/jpm.2023.0159 (PMC10998700; doi:10.1089/jpm.2023.0159)
Supplement: Supplemental data [file Suppl_Appendix.pdf]

Supplements

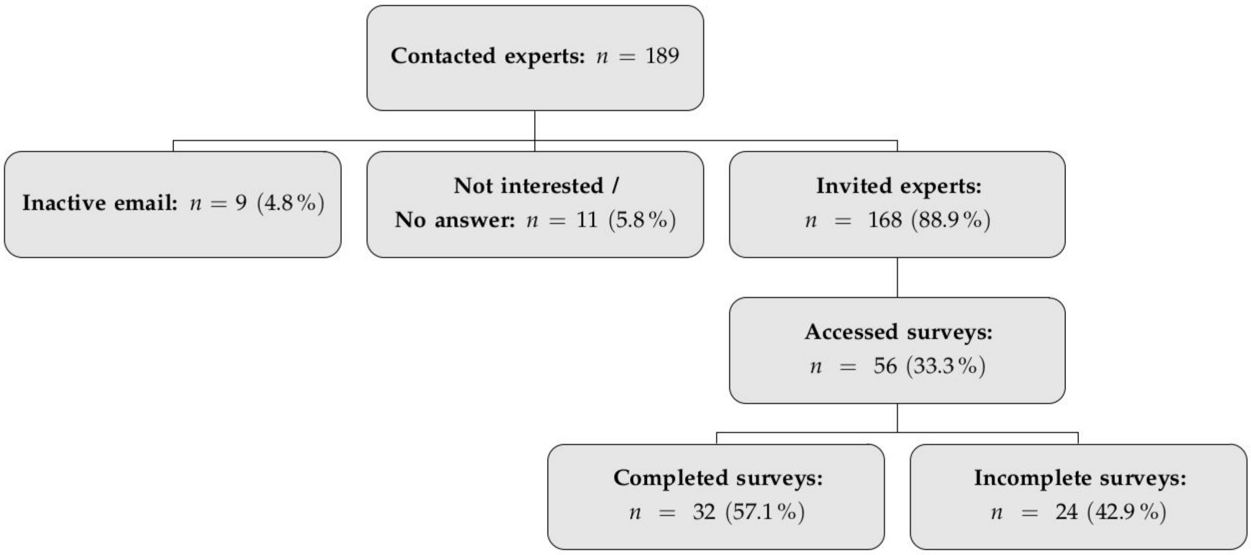

FIGURE 1: FLOW CHART EXPERT GROUP

For Peer Review Only/Not for Distribution

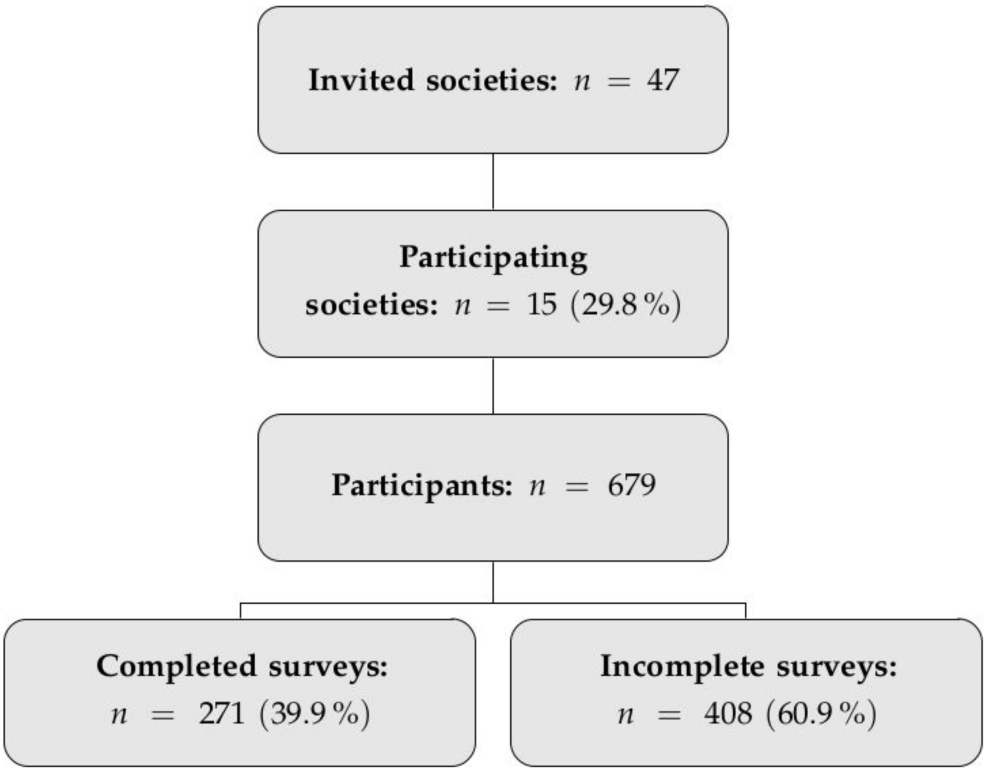

Figure 2: Flow chart EAPC member group

For [illegible] Only/Not for Distribution

For Peer Review Only/Not for Distribution

TABLE 1: PARTICIPATING EAPC ASSOCIATIONS

	Country	Association	Recipients	Completed
1	Italy	Società Italiana di Cure Palliative	6031	63
2	Portugal	Portuguese Association of Palliative Care*		44
3	United Kingdom	International Observatory on End of Life Care	444	28
4	Sweden	Swedish Association for Palliative Medicine*		27
5	Ireland	All Ireland Institute of Hospice and Palliative Care	175	19
6	Denmark	Danish Society of Palliative Medicine	175	18
7	Finland	The Finnish Association for Palliative Care	409	15
8	Poland	Polish Society of Palliative Medicine	21	13
9	Spain	Soc. Española de Cuidados Paliativos	2218	11
10	Belgium	Federation of Palliative Care Flanders	72	10
11	Finland	The Finnish Association for Palliative Medicine	272	9
12	Czech Republic	Czech Society of Palliative Medicine	200	5
13	Czech Republic	Mobile Hospice Forum	21	4
14	Australia	The Australian & New Zealand Society of Palliative Medicine	550	4
				∑ 270

*No information about number of invitations transmitted

Table 2: Socio-demographic results and number of correct answers (max. = 4 per definition, 16 in total, SD = standard deviation, Min-Max = minimum to maximum value)

		Total		Definition 1 (Norway)		Definition 2 (EAPC)		Definition 3 (Japan)		Definition 4 ([x])	
		Mean ± SD	Median (Min-Max)	Mean ± SD	Median (Min-Max)	Mean ± SD	Median (Min-Max)	Mean ± SD	Median (Min-Max)	Mean ± SD	Median (Min-Max)
Gender	Male (N = 86)	7.3 ± 2.66	7 (2–15)	1.8 ± 0.99	2 (0–4)	1.7 ± 1.02	2 (0–4)	1.6 ± 1.08	1 (0–4)	2.3 ± 1.21	2 (0–4)
	Diverse (N = 1)	8.00	8 (8-8)	2.0	2 (2–2)	1.0	1 (1–1)	2.0	2 (2–2)	3.0	3 (3–3)
	Female (N = 213)	7.2 ± 2.64	7 (0-3)	1.7 ± 0.98	2 (0–4)	1.8 ± 1.04	2 (0–4)	1.6 ± 0.98	1 (0–4)	2.1 ± 1.11	2 (0–4)
Setting	Mostly in a hospital setting (N = 161)	7.5 ± 2.59	7 (2–5)	1.7 ± .096	2 (0–4)	1.9 ± 0.99	2 (0–4)	1.6 ± 1.02	1 (0–4)	2.3 ± 1.15	3 (0–4)
	Mostly in a home care setting (N = 122)	7.2 ± 2.69	7 (1–4)	1.8 ± 1.01	2 (0–4)	1.8 ± 1.08	2 (0–4)	1.6 ± 0.98	1.5 (0–4)	2.0 ± 1.1	2 (0–4)
	I don't work in palliative care (N = 16)	5.7 ± 2.52	6 (0–9)	1.0 ± 0.81	1 (0–3)	1.3 ± 0.86	1 (0–3)	1.3 ± 1.08	1.5 (0–3)	2.0 ± 1.26	2 (0–4)
Profession	Physician (N = 203)	7.6 ± 2.64	8 (2–15)	1.8 ± 0.97	2 (0–4)	2.0 ± 1.01	2 (0–4)	1.6 ± 1.00	2 (0–4)	2.2 ± 1.16	2 (0–4)
	Nurse (N = 77)	6.8 ± 2.60	7 (1–13)	1.5 ± 1.00	1 (0–4)	1.5 ± 1.05	1 (0–4)	1.4 ± 1.03	1 (0–4)	2.2 ± 1.10	2 (0–4)
	Psycho-oncologist/psychologist (N = 4)	4.8 ± 2.06	5 (3–7)	1.5 ± 0.58	1.5 (1–2)	1.5 ± 1.00	1 (1–3)	0.8 ± 0.5	1 (0–1)	1.0 ± 0.82	1 (0–2)
	Other (N = 19)	6.4 ± 2.29	6 (0–9)	1.4 ± 0.76	2 (0–2)	1.5 ± 0.9	2 (0–3)	1.4 ± 1.02	2 (0–3)	2.1 ± 1.13	2 (0–4)
Professional Experience in years	0 (N = 4)	6.3 ± 3.10	7 (2–9)	1.3 ± 0.5	1 (1–2)	1.0 ± 0.82	1 (0–2)	1.5 ± 1.29	1.5 (0–3)	2.5 ± 1.91	3 (0–4)
	1–5 (N = 69)	7.3 ± 2.86	8 (1–13)	1.8 ± 1.01	2 (0–4)	1.8 ± 1.11	2 (0–4)	1.7 ± 1.08	2 (0–4)	2.1 ± 1.12	2 (0–4)
	6–10 (N = 66)	7.1 ± 2.70	7 (0–14)	1.7 ± 0.97	2 (0–4)	1.9 ± 0.94	2 (0–4)	1.5 ± 1.06	1 (0–4)	2.1 ± 1.17	2 (0–4)

For Peer Review Only/Not for Distribution

	≥ 11 (N = 163)	7.3 ± 2.54	7 (2–15)	1.7 ± 0.97	2 (0–4)	1.8 ± 1.03	2 (0–4)	1.6 ± 0.95	1 (0–4)	2.2 ± 1.13	2 (0–4)
Experience in PC Research	Yes (N = 172)	7.4 ± 2.44	7 (3–15)	1.8 ± 0.98	2 (0–4)	1.9 ± 1.02	2 (0–4)	1.6 ± 1.00	1 (0–4)	2.1 ± 1.11	2 (0–4)
	No (N = 113)	7.1 ± 2.95	7 (0–13)	1.7 ± 0.99	2 (0–4)	1.7 ± 1.01	2 (0–4)	1.6 ± 1.02	1 (0–4)	2.1 ± 1.18	2 (0–4)
Experience in PC Research on Sedation	Yes (N = 73)	7.2 ± 2.52	7 (2–15)	1.7 ± 1.03	2 (0–4)	1.7 ± 1.12	1 (0–4)	1.7 ± 0.86	2 (0–4)	2.2 ± 1.13	2 (0–4)
	No (N = 210)	7.3 ± 2.72	7 (0–14)	1.7 ± 0.97	2 (0–4)	1.9 ± 0.98	2 (0–4)	1.5 ± 1.05	1 (0–4)	2.1 ± 1.14	2 (0–4)
Group	EAPC Group (N = 271)	7.2 ± 2.64	7 (0–15)	1.7 ± 0.98	2 (0–4)	1.8 ± 1.02	2 (0–4)	1.5 ± 1.02	1 (0–4)	2.2 ± 1.15	2 (0–4)
	Expert (N = 32)	8.1 ± 2.55	8 (3–13)	2.1 ± 0.95	2 (0–4)	2.0 ± 1.11	2 (0–4)	1.8 ± 0.81	2 (0–3)	2.2 ± 1.11	2 (0–4)
Grouped Age	18–25 (N = 1)	8.0	8 (8–8)	1.0	1 (1–1)	1.0	1 (1–1)	2.0	2 (2–2)	4.0	4 (4–4)
	26–44 (N = 89)	7.6 ± 2.71	8 (1–14)	1.8 ± 0.99	2 (0–4)	1.9 ± 0.99	2 (0–4)	1.6 ± 1.08	2 (0–4)	2.3 ± 1.17	2 (0–4)
	≥ 45 (N = 209)	7.1 ± 2.59	7 (0–15)	1.7 ± 0.98	2 (0–4)	1.8 ± 1.05	2 (0–4)	1.5 ± 0.97	1 (0–4)	2.1 ± 1.12	2 (0–4)

For Peer Review Only Not for Distribution

Norwegian definition applied to case 2			
	Answer: Yes, fits the definition	Answer: Not sure whether it fits	Answer: No, doesn't fit the definition
**Category**	**This is a case of bad practice!**		
**Subcategory**	**[unspecified]**		
	**N = 5**	**N = 1**	**N = 4**
Example	»It is PS, but it is incorrectly performed«	»Yes it is palliative sedation by intention. But the procedure is badly conducted.«	»this is a case of misuse of sedativa«
**Subcategory**	**… – it was carried out in an unethical way**		
	**N=2**		
Example	»It is palliative sedation. But it is no legal in this case and it is not ethical correct.«	–	–
**Subcategory**	**… – family's or patient's informed consent was or may have been missing**		
	**N=16**	**N=6**	**N=24**
Example	»It meets the definition, but since the patient was not informed, the treatment isn't justified.«	»There should be a consent with the patient!«	»because the patient did not consent«
**Category**	**Symptoms were not or may not have been refractory**		
	**N=5**	**N=4**	**N=16**
Example	»the physician incorrectly felt that suffering could not be relieved any other way, intending to treat his suffering«	»I'm not sure that everything as been done to alleviate the suffering«	»I think the suffering could have been attempted to be alleviated in other ways«
**Category**	**More information is necessary to come to a decision:**		
**Subcategory**	**Prognosis**		
		**N=2**	
Example	–	»Life expectancy is not specified«*	–
**Category**	**I have a comment on the definition**		
**Subcategory**	**The definition is inadequate**		
	**N=6**		**N=1**
Example	»Although this definition is lacking clarity around goals of care, communication, and consent.« (U274)	–	»in the conduct of the doctor there is no offer of psychological and spiritual support so that the patient's depressive state cannot be defined as refractory simply because of the ineffectiveness of antidepressant drug therapy. The definition lacks the requirement of the patient's consent which must be requested where possible and appropriate«
**Category**	**It's another kind of treatment**		
**Subcategory**	**It's euthanasia**		
			**N=5**
Example	–	–	»in this case if we discontinue hydration and nutrition (and the patient is not dying in hours or days) is not PS but euthanasia«

*** translated with Deepl**

TABLE 3: NORWEGIAN DEFINITION APPLIED TO VIGNETTE 2

EAPC definition applied to case 2			
	**Answer: Yes, fits the definition**	**Answer: Not sure whether it fits**	**Answer: No, doesn't fit the definition**
**Category**	**This is a case of bad practice!**		
**Subcategory**	**[unspecified]**		
			**N = 5**
Example	–	–	»No, because the guidelines were not followed«
**Subcategory**	**… – it was carried out in an unethical way**		
	**-**	**N = 2**	**N = 16**
Example	–	»Maybe this was not ethically acceptable?«	»The case isn't ethically acceptable so the definition does not apply«
**Subcategory**	**… – family's or patient's informed consent was or may have been missing**		
	**N = 2**	**N = 6**	**N = 29**
Example	»Yes, but it should be of course with the necessary information of patient and family..«	»Palliative sedation has to be initiated in cooperation with the patient, which was not the case here.«	»This was done without consent or discussion«
**Subcategory**	**There wasn't enough monitoring**		
			**N = 1**
Example	–	–	»The procedure is not monitored for adverse reactions, only for absent awareness. Too deep sedation will lead to death. Depression is not an intractable suffering, it is treated by psychiatrists. Existential suffering is also not an intractable suffering.«
**Category**	**Symptoms were not or may not have been refractory**		
			**N = 5**
Example	–	–	»1) Same answer as above [»There has been no consultation with other experts to confirm that this is a refractory state.«] 2) It is anything but ethical: no consultation with the patient, his family and the rest of the team, no discussion about hydration. 3) Starting with 5mg/h of midazolam seems to me to be far too high, as the aim should be to find the minimum dose necessary to relieve suffering.«
**Category**	**More information is necessary to come to a decision:**		
**Subcategory**	**I need more information about the monitoring**		
		**N = 1**	
Example		»Monitoring is not described sufficiently«	
**Category**	**I have a comment on the definition**		
**Subcategory**	**It's the best definition because it includes ethical aspects**		
			**N = 1**
Example			»The best definition because of including the ethical aspect of application«
**Subcategory**	**It's not necessary to include acceptability for the health care providers**		
**N=**	**N = 1**		
Example	»Ethically acceptable to the patient and family is enough to me, the "team" hopefully has the correct "helping them" attitude....«	–	–
**Category**	It's another kind of treatment		
**Subcategory**	**It's continuous deep sedation**		
			**N = 1**
Example			»It is continuous deep sedation«

*** translated with Deepl**

TABLE 4: EAPC DEFINITION APPLIED TO VIGNETTE 2

EAPC definition applied to case 3			
	**Answer: Yes, fits the definition**	**Answer: Not sure whether it fits**	**Answer: No, doesn't fit the definition**
**Category**		**This is a case of bad practice!**	
**Subcategory**		**[unspecified]**	
			**N = 1**
**Example**	–	–	»This is bad care«
**Subcategory**		**… – family's or patient's informed consent was or may have been missing**	
	**N = 1**	**N = 1**	**N = 9**
**Example**	»It seems to be the almost the "perfect marriage" between the definition and the vignette. The absence of any further illness modifying therapy, the awareness of his own condition, the fact of the family has been consulted make this case fits to the vignette, except for the fact that the patient seemed not to be involved in the decision making process.«	»There is no talk of sharing with the patient who is aware and asks what can be done«*	»No mention of a discussion about sedation versus new analgesic with the patient«
**Subcategory**		**Medication was not titrated**	
		**N = 1**	
Example	–	»Palliative sedation should also be titrated to the lowest effective dosage.«	–
**Category**		**Symptoms were not or may not have been refractory**	
		**N = 5**	**N = 12**
Example	–	»"Otherwise intractable" may not be true in this case, since alternative medication has not been used«	»Because there was an alternative, not necessarily intractable.«
**Category**		**More information is necessary to come to a decision:**	
**Subcategory**		**It's not clear whether informed consent was given**	
		**N = 1**	
Example	–	»It's necessary to know if the patient has been asked for sedation before the start of the midazolam«	–
**Category**		**I have a comment on the definition**	
**Subcategory**		**"Regular monitoring" should be clarified in the definition**	
		**N = 1**	
Example		»The case does not clearly state that the physician/ team intent or pt agreement was to induce a reduced level of consciousness. However, the dosing of Midazolam at 4mg/h suggests that it was the physician intent. Note: Clarity regarding what is meant by 'regular monitoring' (and its acceptability to the pt and family) is still needed in the proposed definitions.«	
**Subcategory**		**The treatment is not defined as a last option**	
		**N = 1**	
Example	»Nothing is said here about the last option, so I would agree here.«*	–	–
**Category**		**This is a case of correct practice**	
**Subcategory**		**It was carried out according to the patient's wishes**	
	**N = 2**		
Example	»Having respected the patient's wishes, sedation carried out by the treating team falls within the proposed definition«	–	–
**Subcategory**		**The patient was monitored**	
	**N = 2**		
Example	»All was monitored«	–	–

*** translated with Deepl**

TABLE 5: EAPC DEFINITION APPLIED TO VIGNETTE 3

[redacted] definition applied to case 4			
	Answer: Yes, fits the definition	Answer: Not sure whether it fits	Answer: No, doesn't fit the definition
**Category**		**More information is necessary to come to a decision:**	
**Subcategory**		**It's not clear whether informed consent was given**	
		**N = 1**	
Example	–	»Before starting Midazolam infusion, pt and family wanted to maintain pt being able to communicate, thus avoiding sedation. Piece missing: was there further discussion with pt/ family before the Midazolam dose was increased to 2mg/h? Was there a new defined consensus treatment goal?Physician intent with dose increase to 2mg/h not given - (with Midazolam dose of 2mg/h, I would have inferred that the physician intent was to sedate).«	–
**Subcategory**		**I need information about the cause of the patient's symptoms (n = 1)**	
		**N = 1**	
Example	–	»We need to understand what is causing delirium«	–
**Subcategory**		**I need information about the reason for the change in medication (n = 1)**	
		**N = 1**	
Example	–	»Reason for dose increase«	–
**Subcategory**		**I need information about the point of reference (n=1)**	
		**N = 1**	
Example	–	»Difficult to make sense of your case because at what point are you asking me to judge? During sedation or when it is withdrawn?«	–
	–	–	–
**Category**		**It was an unsuccessful attempt**	
	**N = 3**	**N = 1**	**N = 4**
Example	»Yes, but did not work out«	»Unsuccesfull attempt at PS«	»It was the aim to achieve this but was not achieved. Suffering was not relieved but increased and sedation was not actually achieved.«
		**A different intention is described in the case**	
			**N = 3**
Example	–	–	»Sedation was not the goal.«

*** translated with Deepl**

TABLE 6: [X] DEFINITION APPLIED TO VIGNETTE 4
